# Functional Outcome of Radius and Ulnar Shaft Fractures Treated With Intramedullary Kirschner’s Wire Fixation in Children

**DOI:** 10.7759/cureus.89456

**Published:** 2025-08-05

**Authors:** Haroon Yousaf, Shafqat Ullah Khan, Muhammad Naqqash, Mubbshir Khan, Bilal Ahmad, Atizaz A Jan, Maria Ahmad

**Affiliations:** 1 Trauma and Orthopaedics, Queen Elizabeth Hospital, Birmingham, GBR; 2 Trauma and Orthopaedics, Mardan Medical Complex, Mardan, PAK; 3 Accident and Emergency Department, District Headquarter Hospital, Lakki Marwat, PAK; 4 Trauma and Orthopaedics, University Hospitals Birmingham, Birmingham, GBR; 5 Trauma and Orthopaedics, Hayatabad Medical Complex Peshawar, Peshawar, PAK; 6 Trauma and Orthopaedics, Saidu Group of Teaching Hospitals, Swat, PAK; 7 Trauma and Orthopaedics, Khyber Teaching Hospital, Peshawar, PAK; 8 Trauma and Orthopaedics, University Hospital Crosshouse, Kilmarnock, GBR; 9 Diagnostic Radiology, Mardan Medical Complex, Mardan, PAK

**Keywords:** fractures, functional outcome, kirschner wire fixation, radius shaft fracture, ulnar shaft fracture

## Abstract

Introduction: Fractures are a common occurrence in childhood, with approximately one-third of boys and girls sustaining at least one fracture before the age of 17. Both-bone forearm fractures, particularly those involving the radius and ulna, are more common in the non-dominant hand and in boys and usually involve the distal portions of both bones. If not properly treated, these injuries can have a significant impact on limb function.

Objective: This study aimed to determine the functional outcomes of radius and ulnar shaft fractures in children treated with intramedullary Kirschner’s wire fixation, assessed using standardized, objective clinical criteria at eight weeks postoperatively, and to analyze associated demographic and fracture-related factors and postoperative complications.

Materials and methods: This descriptive study was conducted in the Department of Orthopedic Surgery at Bacha Khan Medical College/Mardan Medical Complex, Mardan, over a seven-month period from March 8, 2024, to October 8, 2024. During this time, a total of 348 pediatric patients aged four to 14 years presented with diaphyseal forearm fractures, of whom 209 were successfully managed non-operatively with closed reduction and casting. The remaining 139 children (both genders), whose fractures were deemed unstable after attempted closed reduction - defined radiographically as angulation >10°, displacement >50%, or rotational malalignment - were included in this study. Both open and closed fractures meeting these criteria were eligible. Functional outcomes following intramedullary Kirschner wire fixation were systematically assessed at an eight-week postoperative follow-up. Outcomes were categorized as excellent, good, fair, or poor based on objective clinical assessment, which included measurement of elbow and forearm range of motion, evaluation of pain, presence of residual deformity, and ability to return to age-appropriate daily activities, using standardized institutional criteria.

Results: The mean age of the participants was 8.75 ± 2.97 years. Regarding functional outcomes, 99 children (71.2%) achieved an excellent result, 20 (14.4%) had a good outcome, 13 (9.4%) had a fair outcome, and seven (5%) had a poor outcome. Functional outcomes were slightly better in younger children (aged four to nine years) and in males, although the differences were not statistically significant. No major postoperative complications, defined as those requiring reoperation or resulting in permanent functional impairment, were observed during the eight-week follow-up. Minor complications such as transient pin tract infections and minor wire migration were noted but resolved without additional surgery.

Conclusion: Intramedullary Kirschner wire fixation proved to be an effective treatment for unstable radius and ulnar shaft fractures in children, resulting in predominantly favorable functional outcomes. The majority of patients demonstrated excellent recovery with minimal complications, supporting the procedure’s reliability and safety in the pediatric population. Outcomes were consistent across age groups and genders, with slightly better results observed in younger children and those with early intervention. Future multicenter studies with larger sample sizes and longer-term follow-up are recommended to validate these findings and further refine treatment protocols for pediatric forearm fractures.

## Introduction

Fractures of the radius and ulna shafts are among the most common long bone injuries in children, accounting for approximately 5-10% of all pediatric fractures [1]. These injuries typically occur as a result of falls, sports activities, or trauma and can lead to significant functional impairment if not managed appropriately [2]. Due to the crucial role of the forearm in upper limb rotation and hand positioning, proper anatomical alignment and early mobilization are essential to ensure optimal functional recovery in the pediatric population [3].

Traditionally, non-operative management with closed reduction and casting has been the mainstay of treatment for pediatric forearm fractures [4]. While this approach is successful in the majority of cases, certain situations, such as unstable fractures, failure of closed reduction, open fractures, and those with associated neurovascular injury, require surgical intervention [5]. In recent years, internal fixation methods have gained popularity for managing these complex cases [6]. Among these methods, elastic stable intramedullary nailing (ESIN) and plate osteosynthesis are widely used, offering reliable outcomes but with certain limitations, including soft tissue dissection, risk of infection, longer operative time, and cost implications [7].

Intramedullary fixation using Kirschner's wires (K-wires) presents an alternative surgical option that is minimally invasive, cost-effective, and technically less demanding [8]. K-wire fixation involves inserting smooth stainless-steel wires into the medullary canal of the radius and ulna to maintain fracture alignment while allowing early mobilization [9]. This method is particularly appealing in resource-limited settings due to its affordability, shorter operative time, and reduced surgical morbidity. Several studies have reported encouraging outcomes with intramedullary K-wire fixation, with union rates exceeding 95% and satisfactory range of motion in over 85% of cases, alongside low rates of major complications such as malunion or nonunion [10,11,12]. Al-Moaish et al. [12] observed excellent or good functional outcomes in 60.9% of pediatric patients treated with intramedullary K-wires, while Shams et al. [13] reported a union rate of 97% with minimal soft tissue complications. However, concerns persist regarding rotational instability, risk of wire migration, and potential injury to growth plates, necessitating further evaluation of its long-term functional outcomes [4,8,14].

Despite the growing clinical use of intramedullary K-wire fixation, there remains limited consensus on its efficacy compared to other surgical modalities, particularly in terms of long-term functional outcomes in children [15]. Most existing literature focuses on short-term radiological healing and complication rates, with few studies providing comprehensive assessments of postoperative limb function using standardized scoring systems [16]. There is a lack of robust data specifically evaluating the functional outcomes of radius and ulna shaft fractures treated with intramedullary K-wire fixation in pediatric patients, especially in resource-constrained settings where this technique is frequently employed for its affordability and lower morbidity.

Therefore, the present study was designed to address this gap by systematically evaluating the clinical and functional outcomes of unstable, displaced diaphyseal fractures of the radius, ulna, or both bones in children treated with intramedullary Kirschner’s wire fixation. The study aimed to determine the proportion of patients achieving excellent, good, fair, or poor functional recovery at eight weeks, based on objective clinical assessment of range of motion, pain, deformity, and return to daily activities. In addition, it sought to explore associations between functional outcomes and demographic or fracture-related factors, to document the frequency and nature of postoperative complications, and to compare these findings with existing literature in order to contribute to evidence-based recommendations for managing pediatric forearm fractures.

Objective

This study aimed to determine the functional outcomes of radius and ulnar shaft fractures in children treated with intramedullary Kirschner’s wire fixation, assessed using standardized, objective clinical criteria at eight weeks postoperatively, and to analyze associated demographic and fracture-related factors as well as postoperative complications.

## Materials and methods

Study setting

This study was conducted at the Department of Orthopedic Surgery, Bacha Khan Medical College, Mardan Medical Complex, Mardan.

Study design and duration

We conducted a descriptive study over a fixed period of seven months, from March 8, 2024, to October 8, 2024. Since the study was time-bound, a non-probability convenience (consecutive) sampling technique was appropriately employed to include all eligible patients presenting during the study period. During the study period, a total of 348 pediatric patients aged four to 14 years presented with diaphyseal forearm fractures. Of these, 209 (approximately 60%) were successfully managed with closed reduction and casting alone. The remaining 139 patients (40%) met the inclusion criteria of instability after attempted closed reduction and were included in the study cohort.

Sample size

A minimum sample size of 138 patients was estimated using the WHO sample size calculator, assuming a 10% proportion of poor functional outcome, 95% confidence level, and 5% margin of error. However, the final number of 139 was based on the total eligible cases presenting within the study period.

Inclusion and exclusion criteria

During the study period, 348 pediatric patients aged four to 14 years presented with diaphyseal forearm fractures. Of these, 139 (40%) had unstable fractures after attempted closed reduction, defined as angulation >10°, displacement >50%, or rotational malalignment on post-reduction radiographs, and were included in the study. Both open and closed fractures meeting these criteria were eligible. While many younger children (four to nine years) typically have sufficient remodeling potential for nonoperative care, we specifically included those whose fractures remained unstable after reduction, representing a clinically significant subset warranting surgical stabilization.

We excluded patients with fractures involving the metaphyseal-diaphyseal junction of the elbow or wrist, pathological fractures, associated radial head fractures, Monteggia or Galeazzi injuries, concomitant visceral injuries, or those managed conservatively (casting alone) during the same period. The age range of four to 14 years was chosen to include skeletally immature children beyond the toddler age group, where remodeling potential is often adequate, and below the typical age of skeletal maturity, to avoid confounding from adult-type fracture patterns and fixation requirements.

Ethical considerations

The study protocol was approved by the Institutional Review Board of Medical Teaching Institution - Bacha Khan Medical College, Mardan (approval no. 392/BKMC). Informed consent was obtained from parents or guardians for all participants. In addition, verbal assent was sought from children above eight years of age, in line with ethical standards for pediatric research.

Data collection procedure

Each child underwent detailed clinical examination and radiographs (anteroposterior and lateral views of the forearm) to confirm diagnosis and assess fracture location (proximal, middle, or distal third) and bone(s) involved. Sociodemographic data (age, sex, residence, father’s profession, mother’s education, and household income) and fracture characteristics (pattern, type, location, open/closed, and duration since injury) were recorded. The maternal profession was not recorded, as the majority of mothers in our study population were homemakers. Surgical details, including the criteria for K-wire diameter selection (typically ~40% of the narrowest medullary canal diameter) and intraoperative findings, were documented.

Functional outcome assessment

Functional outcomes were assessed during follow-up based on objective clinical examination, focusing on ROM at the elbow, wrist, and forearm (pronation/supination), the presence and severity of pain, residual deformity, and the child’s ability to perform age-appropriate daily activities.

“Return to function” was defined as the resumption of routine school and play activities without limitations, confirmed through both clinical observation and parent/guardian reports. Activity limitations were evaluated by assessing the child’s ability to perform daily age-appropriate tasks such as writing, dressing, eating, and playing. These assessments were based on a combination of direct clinical examination and structured caregiver feedback during follow-up visits.

Partial limitation was defined as difficulty or slowness in performing some tasks without complete inability. Significant limitation referred to the inability to perform key tasks independently or avoidant behavior due to pain, weakness, or discomfort.

Functional outcomes were categorized as 1) excellent (full ROM of the elbow and forearm, no pain, no visible deformity, and complete return to normal activities), 2) good (mild (<15°) ROM restriction, minimal or no pain, and no significant limitation in daily tasks), 3) fair (moderate (15-30°) ROM loss, intermittent pain, and partial limitation in daily function), and 4) poor (severe (>30°) ROM loss, persistent pain, visible deformity, or significant activity limitation).

Pain was assessed using validated age-appropriate tools: the Visual Analog Scale (VAS) or Faces Pain Scale-Revised (FPS-R) for children over six years, and observational, behavior-based assessment tools for younger children. Pain was categorized as 1) mild (VAS ≤3 or no interference with activities), 2) moderate (VAS 4-6 or intermittent activity limitation), and 3) severe (VAS ≥7 or persistent interference with function).

Radiological healing was confirmed by the presence of bridging callus across at least three cortices and the disappearance of fracture lines on follow-up radiographs.

Surgical technique

All procedures were performed under general anesthesia with tourniquet control, administered by an experienced pediatric anesthesiology team using a standardized protocol. Anesthesia was induced with intravenous agents and maintained with inhalational anesthetics, ensuring adequate muscle relaxation, analgesia, and hemodynamic stability throughout the surgery. Patients were continuously monitored for vital signs, oxygenation, and perfusion.

The surgeries were performed by the same orthopedic team led by a consultant with at least five years of pediatric orthopedic fellowship training. The radius was approached through a small dorsal incision over the distal metaphysis, and an appropriately sized smooth stainless-steel K-wire (approximately 40% of the narrowest medullary canal diameter) was inserted retrograde into the medullary canal up to the radial head. The ulna was approached through a separate mini-incision at the olecranon, and the K-wire was advanced distally toward the ulnar styloid. Wire ends were bent and left protruding outside the skin to facilitate later removal.

After confirming fracture alignment, hemostasis was achieved, and wounds were closed. An above-elbow plaster of Paris (POP) cast was applied, windowed to allow access for wound inspection and dressing changes. Dressings were applied directly to the surgical sites beneath the cast and were routinely changed on the second postoperative day.

Postoperative care and follow-up

The cast was removed at three weeks, and wires were extracted after confirming radiological union. Patients were followed up every two weeks for eight weeks. Functional outcomes and range of motion were recorded at each visit. Complications such as pin tract infections, wire migration, loss of reduction, delayed union, nonunion, or growth disturbances were noted. Minor pin tract infections were treated with local care and antibiotics without wire removal, and these were not categorized as major complications.

Data analysis

Data were analyzed using IBM SPSS Statistics for Windows, version 25.0 (IBM Corp., Armonk, NY). Continuous variables such as age, BMI, and fracture duration were summarized as mean ± standard deviation or median with interquartile range, based on distribution. Categorical variables such as gender, residence, fracture type, and functional outcomes were expressed as frequencies and percentages. Associations of functional outcome with age, gender, fracture type, residence, and socioeconomic status were explored using chi-square tests, with p ≤ 0.05 considered statistically significant.

## Results

The study population (n = 139) predominantly consisted of children aged four to nine years (56.1%), with a mean age of 8.75 years (SD = 2.974). The majority were male (61.2%), and slightly more participants resided in rural areas (51.8%) than in urban areas (48.2%). The average time since the fracture occurred was 3.01 hours. The demographic and descriptive statistics of the study population are summarized in Table 1.

**Table 1:** Demographic and descriptive statistics of the study population (n = 139). Mean ± standard deviation (SD) is reported for normally distributed variables. The Shapiro-Wilk test determines the median and interquartile range (IQR) for non-normally distributed variables.

Variable	Category	Frequency	Percent (%)	Mean ± SD / Median (IQR)
Age (years)	4–9	78	56.10%	8.75 ± 2.97
	9–14	61	43.90%	
Gender	Male	85	61.20%	—
	Female	54	38.80%	
Residence	Rural	72	51.80%	—
	Urban	67	48.20%	
Duration of fracture (hrs)	—	—	—	2.5 (1.5–4.0)
BMI (kg/m²)	—	—	—	17.19 ± 1.97

**Figure 1:**
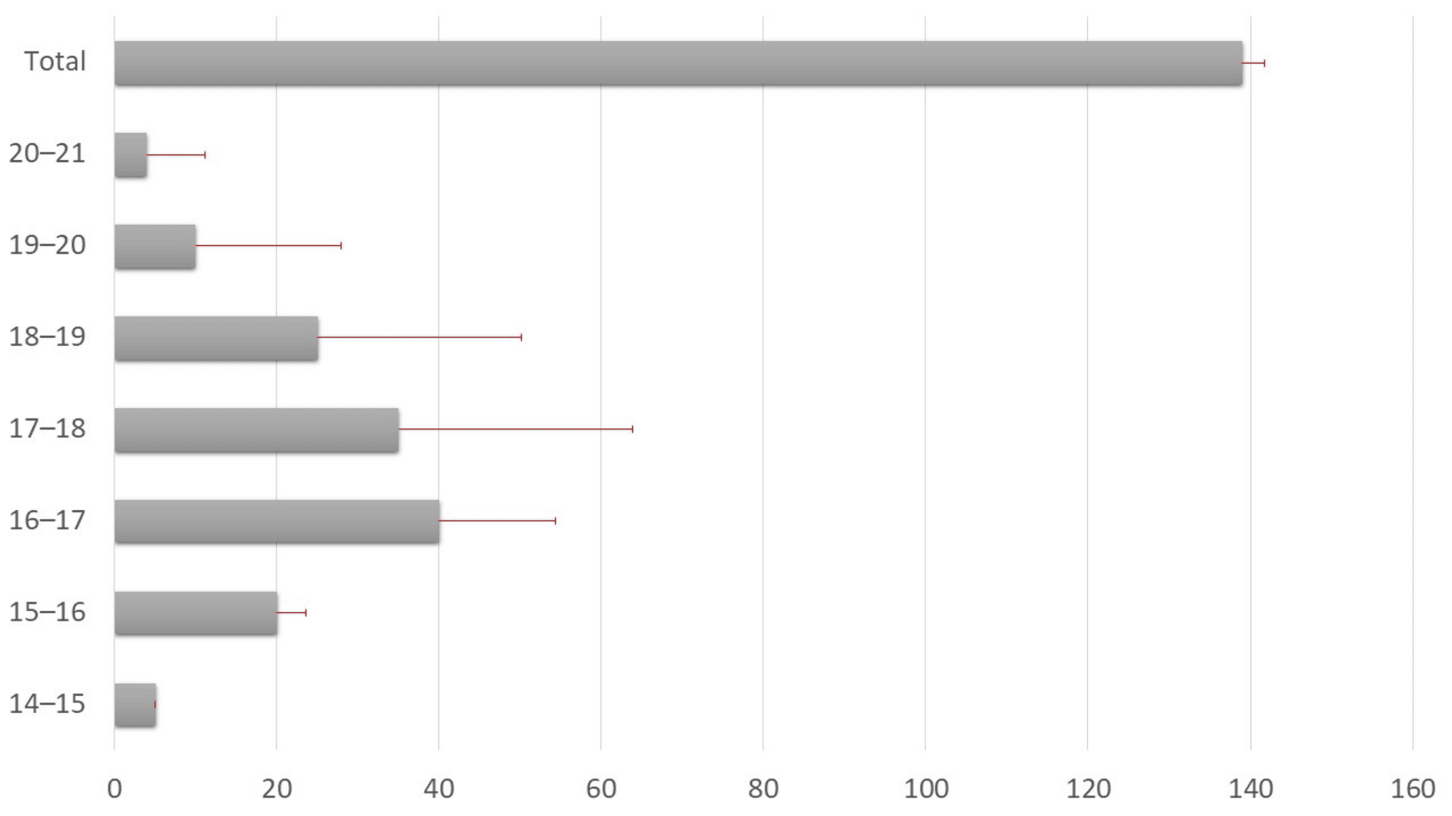
Distribution of BMI among the study participants (n = 139)

The mean BMI of the study group was 17.19 kg/m² (SD = 1.97). The distribution of BMI was examined and found to be approximately symmetric and unimodal, without evidence of significant skewness or bimodality (Figure 1), indicating relatively consistent nutritional status among participants.

Most fathers were businessmen (31.7%), followed by laborers (29.5%) and office workers (23.0%). Maternal education levels were skewed toward secondary education (44.6%), and about one-third had intermediate or higher qualifications. In terms of socioeconomic status, the majority of families (54.7%) fell into the middle-income bracket, while 31.7% belonged to the lower-income group. These characteristics help contextualize the patients’ background, which may have implications for health-seeking behavior and access to timely medical care (as shown in Table 2).

**Table 2:** Socioeconomic and parental characteristics.

Variable	Category	Frequency	Percent
Profession of father	Office job	32	23.0%
	Labor	41	29.5%
	Businessman	44	31.7%
	Other	22	15.8%
Education of mother	Illiterate	31	22.3%
	Secondary	62	44.6%
	Intermediate and above	46	33.1%
Socioeconomic status	Low (<50K PKR/month)	44	31.7%
	Middle (50K–100K PKR/month)	76	54.7%
	High (>100K PKR/month)	19	13.7%

Fractures of the radius were more frequent (60.4%) compared to ulna fractures (46.8%). Functional outcomes were generally favorable, with a significant majority (71.2%) achieving excellent recovery. An additional 14.4% had good outcomes, while only 9.4% and 5.0% were categorized as fair and poor, respectively. The mean functional outcome score, calculated by assigning scores of excellent = 4, good = 3, fair = 2, and poor = 1, was 3.52, indicating that most patients had good-to-excellent outcomes. Fracture pattern categories are mutually exclusive: isolated radius fracture (radius fractured, ulna intact), isolated ulna fracture (ulna fractured, radius intact), and both bones fractured. Among the study population, isolated radius fractures were most common (n = 74, 53.2%), followed by isolated ulna fractures (n = 55, 39.6%), while both bones were fractured in 10 (7.2%) cases. These findings point toward effective management strategies and possibly the benefit of early intervention in this pediatric population (as shown in Table 3).

**Table 3:** Clinical characteristics and functional outcomes. *Functional outcome categories: excellent = full ROM, no pain or deformity, full return to activities; good = <15° ROM loss, minimal/no pain, no significant limitations; fair = 15–30° ROM loss, intermittent pain, partial limitation; poor = >30° ROM loss, persistent pain, deformity, or inability to return to normal activities.

Variable	Category	Frequency	Percent
Type of fracture	Radius: Yes	84	60.4%
	Radius: No	55	39.6%
	Ulna: Yes	65	46.8%
	Ulna: No	74	53.2%
Fracture pattern	Isolated radius fracture	74	53.2%
	Isolated ulna fracture	55	39.6%
	Both bones fractured	10	7.2%
Functional outcome^*^	Excellent	99	71.2%
	Good	20	14.4%
	Fair	13	9.4%
	Poor	7	5.0%

Functional outcomes stratified by age and gender did not show statistically significant differences (p > 0.05). Although the younger age group (four to nine years) and males had slightly higher frequencies of excellent outcomes, the distribution across all categories remained relatively balanced. This suggests that neither age nor gender significantly influenced the recovery trajectory in this study (as shown in Table 4).

**Table 4:** Stratification of functional outcome by age and gender (n = 139). Associations were tested using the chi-square test. Statistical significance was defined as p < 0.05.

Functional outcome	Age 4–9	Age 9–14	Total by age	Male	Female	Total by gender
Excellent	55 (55.6%)	44 (44.4%)	99	62 (62.6%)	37 (37.4%)	99
Good	11 (55.0%)	9 (45.0%)	20	14 (70.0%)	6 (30.0%)	20
Fair	9 (69.2%)	4 (30.8%)	13	6 (46.2%)	7 (53.8%)	13
Poor	3 (42.9%)	4 (57.1%)	7	3 (42.9%)	4 (57.1%)	7
Total	78 (56.1%)	61 (43.9%)	139	85 (61.2%)	54 (38.8%)	139
P-value	0.69			0.39		
χ² value	1.47			2.05		

When stratifying functional outcomes by residence, socioeconomic status, fracture duration, and fracture type, no statistically significant associations were found. However, patients from the middle socioeconomic group and those with fracture durations between one and three hours had slightly better outcomes. Fracture type (radius vs. ulna) also influenced recovery, but differences did not reach statistical significance. These patterns may help inform clinical priorities for early treatment and equitable care across different demographic groups (as shown in Table 5).

**Table 5:** Stratification of functional outcome by residence, socioeconomic status (monthly income in PKR), duration of fracture (in hours), and fracture type (radius and ulna) (n = 139). The chi-square (χ²) test was used. Statistical significance was defined as p < 0.05.

Functional outcome	Rural	Urban	Low SES (PKR)	Middle SES (PKR)	High SES (PKR)	1–3 hours	>3 hours	Radius fracture	No radius fracture	Ulna fracture	No ulna fracture
Excellent	53	46	32	51	16	61	38	58	41	50	49
Good	9	11	8	11	1	10	10	10	10	10	10
Fair	5	8	3	9	1	9	4	10	3	4	9
Poor	5	2	1	5	1	4	3	6	1	1	6
Total	72	67	44	76	19	84	55	84	55	65	74
p-value	0.47		0.62			0.7		0.22		0.17	
χ² value	2.55		2.3			1.4		4.53		5.71	

## Discussion

The present study evaluated the functional outcomes of intramedullary K-wire fixation in pediatric patients with unstable diaphyseal fractures of the radius and/or ulna. The results demonstrated a high rate of excellent outcomes (71.2%) with very few poor results (5.0%), indicating that K-wire fixation is a reliable method for managing such fractures in children. Most patients fell in the four-to-nine-year age group and were male, with no statistically significant differences in outcomes based on age, gender, fracture type, or socioeconomic status.

When compared to existing literature, the demographic trends observed in this study, such as the higher incidence in young males, mirror well-established patterns seen globally. This consistency supports the notion that forearm fractures in children are influenced by behavioral and developmental factors, with boys in the younger age group being more prone due to higher physical activity levels [17]. The predominance of radius fractures also reflects its anatomical vulnerability and has been similarly reported in prior epidemiological studies [18].

The excellent functional outcomes observed align with previous evidence showing that pediatric bone has a strong healing potential, particularly when treated promptly with stable fixation [19]. Studies using elastic intramedullary nailing or plate fixation also report high rates of satisfactory results, but these often come with increased cost, longer operating time, and greater soft tissue disruption [20]. By contrast, K-wire fixation offers a minimally invasive and cost-effective solution with comparable functional outcomes, especially beneficial in resource-limited settings [21].

Rotational instability and growth plate injury are known concerns with K-wire techniques, yet in this study, complication rates were low, and no significant long-term deficits were observed. This may be attributed to the surgical team's experience and adherence to anatomical insertion techniques. Our findings of relatively low complication rates with K-wire fixation are consistent with the notion that outcomes depend heavily on surgical technique, follow-up care, and patient selection. Although other studies have reported higher complication rates with K-wires, variations in technique, follow-up duration, and patient selection likely account for such discrepancies [22].

Al-Moaish et al., in a prospective study of pediatric forearm fractures in Yemen, reported pin site irritation in 34.8% of patients and superficial wound infections in 17.4%, although all resolved with appropriate treatment [12]. Similarly, Sharma et al. documented an overall complication rate of 32.3% in children treated with K-wires for upper extremity fractures, including pin tract infections, wire loosening, and rare neurovascular injuries [23]. By contrast, Smith et al., evaluating K-wire fixation in adult four-corner fusions, found a low nonunion rate of 3.5% and reported no pin-related complications requiring revision surgery [24]. These differences likely reflect variations in patient age, fracture type, and surgical protocols, underscoring the importance of standardized techniques and meticulous post-operative care to minimize complications.

Despite slightly better outcomes being observed in radius-only fractures compared to ulna or combined fractures, the differences were not statistically significant. This contrasts with some existing studies that report delayed healing and increased complication rates in dual-bone injuries [25]. The relatively uniform outcomes in this study may reflect improvements in perioperative care, early mobilization protocols, and uniform follow-up strategies that mitigate traditional disadvantages associated with ulna or complex fractures [26].

Limitations and future suggestions

This study has several limitations. Its single-center, descriptive design and relatively short follow-up period (eight weeks) limit the generalizability and ability to assess long-term outcomes, such as growth disturbances, physeal arrest, or late hardware-related complications. Furthermore, while we defined clear, objective criteria for functional outcome categories (excellent, good, fair, poor) based on range of motion, pain, deformity, and return to daily activities, these categories were not derived from a validated scoring system, which may limit comparability with other studies. Future research should consider incorporating validated and standardized functional assessment tools, such as the Pediatric Outcomes Data Collection Instrument (PODCI) or the child-adapted Disabilities of the Arm, Shoulder, and Hand (QuickDASH-C) questionnaire, to enhance the granularity and reproducibility of results. Socioeconomic data were recorded in detail, but maternal education alone was used as a proxy for socioeconomic status, based on its recognized relevance in the Pakistani cultural context. However, paternal education and other indicators of household status were not explicitly analyzed, which could provide a more nuanced understanding in future studies.

In addition, the convenience (consecutive) sampling of patients presenting during the defined seven-month study period may introduce selection bias. Future studies should aim for larger, multicenter cohorts with more diverse populations and longer follow-up to strengthen external validity. Finally, randomized controlled trials comparing K-wire fixation directly with alternative surgical or nonoperative approaches, and evaluating the role of structured postoperative rehabilitation programs, are recommended to further optimize management strategies for pediatric forearm fractures.

## Conclusions

In this single-center, short-term study, pediatric forearm fractures, particularly those involving the radius, were more frequently observed in younger male children and were generally associated with excellent short-term functional outcomes when managed promptly with intramedullary Kirschner wire fixation. No significant differences in recovery were observed based on age, gender, socioeconomic status, or residence, suggesting comparable outcomes across these subgroups within our study population. These observations support the effectiveness of the current management approach in similar clinical settings and underscore the potential benefits of early intervention in optimizing recovery in pediatric forearm fractures.
